# Transcriptome Signature of Vγ9Vδ2 T Cells Treated With Phosphoantigens and Notch Inhibitor Reveals Interplay Between TCR and Notch Signaling Pathways

**DOI:** 10.3389/fimmu.2021.660361

**Published:** 2021-08-30

**Authors:** Ayush Madhok, Sajad Ahmad Bhat, Chinna Susan Philip, Shalini Kashipathi Sureshbabu, Shubhada Chiplunkar, Sanjeev Galande

**Affiliations:** ^1^Centre of Excellence in Epigenetics, Department of Biology, Indian Institute of Science and Education and Research (IISER), Pune, India; ^2^Advanced Centre for Treatment, Research and Education in Cancer (ACTREC), Tata Memorial Centre, Navi Mumbai, India; ^3^Homi Bhabha National Institute (HBNI), Mumbai, India; ^4^Department of Life Sciences, School of Natural Sciences, Shiv Nadar University, Greater Noida, India

**Keywords:** γδ T cells, transcriptome, γδ activation, HDMAPP, Notch signaling, TCR signaling, IPP

## Abstract

Gamma delta (γδ) T cells, especially the Vγ9Vδ2 subtype, have been implicated in cancer therapy and thus have earned the spotlight in the past decade. Although one of the most important properties of γδ T cells is their activation by phosphoantigens, which are intermediates of the Mevalonate and Rohmer pathway of isoprenoid biosynthesis, such as IPP and HDMAPP, respectively, the global effects of such treatments on Vγ9Vδ2 T cells remain elusive. Here, we used the high-throughput transcriptomics approach to elucidate the transcriptional changes in human Vγ9Vδ2 T cells upon HDMAPP, IPP, and anti-CD3 treatments in combination with interleukin 2 (IL2) cytokine stimulation. These activation treatments exhibited a dramatic surge in transcription with distinctly enriched pathways. We further assessed the transcriptional dynamics upon inhibition of Notch signaling coupled with activation treatments. We observed that the metabolic processes are most affected upon Notch inhibition *via* GSI-X. The key effector genes involved in gamma–delta cytotoxic function were downregulated upon Notch blockade even in combination with activation treatment, suggesting a transcriptional crosstalk between T-cell receptor (TCR) signaling and Notch signaling in Vγ9Vδ2 T cells. Collectively, we demonstrate the effect of the activation of TCR signaling by phosphoantigens or anti-CD3 on the transcriptional status of Vγ9Vδ2 T cells along with IL2 stimulation. We further show that the blockade of Notch signaling antagonistically affects this activation.

## Introduction

γδ T cells are a small subgroup of T cells, accounting for about 1%–5% of peripheral T-cell populations (1). γδ T cells express different T-cell receptors (TCRs)—γ and δ chains—unlike the classical αβ T cells (2). In further contrast, γδ T cells show aspects of both innate and adaptive immune responses (3) and are considered to bridge the two host-defense mechanisms (4). γδ T cells characteristically are different from their αβ counterparts not only in their TCR usage but also in their tissue localization and MHC-independent antigen recognition (5, 6). The predominantly found γδ T cells in circulating blood are of Vγ9Vδ2 subtype, which have been widely studied in responses against microbial pathogens and cancer in humans (7). Furthermore, Vγ9Vδ2 T cells show dramatic plasticity, antigen presentation, and abundant inflammatory-cytokine production (8). In light of these features, Vγ9Vδ2 T cells are being studied extensively for cancer immunotherapy.

Human γδ T cells get activated and proliferate in response to non-peptidic compounds derived from pathogenic microbes including *Mycobacterium tuberculosis* (9–11). γδ T cells also recognize non-peptide phosphoantigens produced *via* mevalonate pathway such as isopentenyl pyrophosphate (IPP) (12). A similar naturally occurring bacterial metabolite, hydroxyl dimethylallyl pyrophosphate (HDMAPP, also known as HMBPP), is one of the strongest stimulants for Vγ9Vδ2 T cells (13). It has been shown that there is no absolute necessity of antigen presentation through antigen-presenting cells (APCs) or antigen display *via* major histocompatibility complex (MHC) for phosphoantigen-mediated activation of Vγ9Vδ2 T cells (12, 14). Butyrophillin (BTN) family members BTN3A1 and BTN2A1 play crucial roles in phosphoantigen sensing, activation, and proliferation of Vγ9Vδ2 cells (15, 16).

The antitumor effect of γδ T cells is achieved by their virtue to produce proinflammatory cytokines interferon-γ (IFN-γ) and tumor necrosis factor-α (TNF-α), which act in cohort with other factors to induce antitumor immunity and inhibition of cancer angiogenesis (1, 17). Activated γδ T cells also produce cytolytic proteins Granzyme B and Perforin, through which they lyse the tumor cells after migrating to the tumor microenvironment (18). In some tumors, upon hyperactivation of the mevalonate pathway, IPP is overproduced, and activation of Vγ9Vδ2 T cells by IPP is dependent on the transmembrane butyrophillin molecules (15, 19, 20). Sensitivity of these tumors to lysis by Vγ9Vδ2 T cells increases upon treatment with aminobisphosphonates, which leads to accumulation of intracellular IPP (21, 22). We and others have previously shown that prior treatment of cancer cells with zoledronate, an aminobisphosphonate, can greatly increase the efficiency of lysis by activated Vγ9Vδ2 T cells (23, 24).

Notch signaling has been extensively characterized in immune development and differentiation, and their maintenance and activation (25, 26). It is essential for early T cell fate choice and αβ *vs* γδ lineage diversification. Notch signaling is also shown to promote antitumor activity of T cells and NK cells (27). Our earlier results demonstrated that Notch expression in γδ T cells is mediated by TCR activation, and inhibition of γ-secretase, which cleaves Notch for nuclear export, leads to dramatic reduction in cytolytic activity of activated γδ T cells (28).

Multiple transcriptomics and genomics studies have provided insights of the spatiotemporal control of T-cell activation, differentiation, and development—especially in the context of CD4^+^ and CD8^+^ αβ subsets (29–31). Recently, numerous single-cell genomic studies have provided knowledge about the finer distinctions of T-cell functionality at a single-cell resolution and the heterogeneity among marker-based sorted populations (32–37). A comprehensive blood single-cell transcriptomics revealed that human TCR Vδ1 and TCR Vδ2 γδ T cells share cytotoxic hallmarks of both CD8 and NK cells but form distinct clusters (38).

Despite growing literature on the antitumor potential of γδ T cells (39), it is still unclear how activation *via* phosphoantigens or anti-CD3 antibodies mediates the effector functions of Vγ9Vδ2 T cells at the molecular level. Here, we performed RNA-sequencing (RNA-seq) of Vγ9Vδ2 T cells with multiple combinations of activating or repressive treatments and elucidated the primary transcriptional pathways utilized in each case. Our analyses revealed key transcription factors (TFs) or their primary pathways that are affected *via* the activation/repression. This study provides important cues towards designing better combinations of target-specific molecules along with the current γδ T cell-based immunotherapies.

## Materials and Methods

### γδ T Cell Separation From Peripheral Blood

Blood samples were collected from three healthy volunteers, and peripheral blood mononuclear cells (PBMCs) were isolated using Ficoll–Hypaque (Sigma -Aldrich) differential density gradient centrifugation. The study was approved by the Institutional Ethics Committee, and written informed consent was obtained from the healthy volunteers before collection of blood samples. Blood samples were processed as per the guidelines of the Institute Biosafety Committee. All experimental procedures involving clinical samples were handled in biosafety cabinets, and laboratory personnel handling blood samples were vaccinated against hepatitis B. γδ T cells were purified from PBMCs using anti-TCR γδ microbeads (clone 11F2; Miltenyi Biotech, Germany) by positive selection, as per the manufacturer’s instructions.

### Purity of Isolated Vγ9Vδ2 T Cells

Flow cytometry analysis of immunomagnetically isolated γδ T cells was performed to ascertain their purity. Briefly, a fraction of isolated γδ T cells were washed with 1× phosphate-buffered saline (PBS), cold fixed with 1% paraformaldehyde for 15 min at 4°C and stained for cell surface markers using the following conjugated antibodies procured from BD Biosciences: Vδ2 TCR-FITC (clone B6), CD56-APC R-700 (clone NCAM16.2), αβ TCR-PE (clone T10B9.1A-31), CD14-PE (clone M5E2), CD11b PE-CF594 (clone ICRF44), CD19-BV786 (clone SJ25C1), Vδ2 TCR-PE (clone B6), and Vδ1 TCR-PerCP-Vio 700 (clone REA173). The cells were incubated for 30 min at 4°C and washed with FACS buffer. The purity and distribution of Vδ2 and Vδ1 populations in isolated γδ T cells were confirmed by flow cytometry using FACS Aria III flow cytometer (BD Biosciences, USA) and analysis performed using FlowJo software (TreeStar, Ashland, USA). The purity of γδ T cells isolated from peripheral blood of three healthy individuals was 96.4 ± 1.07%. As the distribution of Vδ2 population was higher (88.2 ± 5.92%) in the isolated γδ T cells as compared to Vδ1 (7.03 ± 1.26%), these are henceforth referred to as Vγ9Vδ2 T cells (**Supplementary Figure S1**).

### Cell Culture and Treatments

Vγ9Vδ2 T cells were cultured in Roswell Park Memorial Institute (RPMI) 1640 (Invitrogen) supplemented with 10% heat-inactivated AB serum, 2 mM glutamine (Invitrogen), and penicillin–streptomycin (Sigma-Aldrich). Briefly, 1 × 10^6^ Vγ9Vδ2 T cells were seeded in triplicate sets in 24-well flat bottom plates (Nunc) and were treated in various combinations: unstimulated Vγ9Vδ2 T cells, Vγ9Vδ2 T cells stimulated with 50 IU/ml rIL2 (Peprotech), 50 IU/ml rIL2 + HDMAPP (1 nM; Echelon), 50 IU/ml rIL2 + IPP (40 μM; Sigma-Aldrich), 50 IU/ml rIL2 + plate-bound anti-CD3 monoclonal antibody (clone OKT3; BD Biosciences, USA), rIL2 + HDMAPP + γ-secretase inhibitor-X, L-685,458 (GSI-X, 15 μM) (Calbiochem, La Jolla, CA, USA), rIL2 + IPP + GSI-X, and rIL2 + anti-CD3 + GSI-X, using previously standardized concentrations. After 72 h, the viability of Vγ9Vδ2 T cells was determined by Trypan Blue cell exclusion assay. The viability ranged from 86% to 90% for untreated Vγ9Vδ2 T cells and from 93.4% to 94.8% for all other treatments previously mentioned. The harvested cells were snap-frozen in TriZol (Invitrogen) and stored at −80°C for library preparation.

### RNA Isolation, Library Preparation, and Sequencing

Total RNA was isolated using TriZol method. Quantitation of RNA was done on Qubit 4 Fluorometer (Thermo Scientific #Q33238) using RNA HS Kit (Thermo Scientific #Q32852). RNA integrity number (RIN) value was checked using RNA IQ assay (Themo Scientific #Q33222) followed by running on 2100 Bioanalyzer (Agilent #G2939BA). All samples had a RIN value above 8. Five hundred nanograms of total RNA was used to prepare libraries using TruSeq Stranded Messenger RNA (mRNA) Sample Prep Kit (Illumina #20020594), with the TruSeq Stranded mRNA Sample Preparation Guide, Part #15031047. The libraries were sequenced using Illumina HiSeqX platform (Illumina, CA, USA). The read length was 150 bp, paired end. Sequencing depth ranged from 40 to 70M reads.

### Quality Control and Read Mapping

Initial quality of each sample was performed using FastQC v. 0.11.5 (http://www.bioinformatics.babraham.ac.uk/projects/fastqc). Low-quality sequences and adapters were removed using Trimmomatic v. 0.39 (40). The following parameters were used for trimming: ILLUMINACLIP : TruSeq3-SE:2:30:10 LEADING:3 TRAILING:3 SLIDINGWINDOW:4:15 MINLEN:36. More than 98% of paired-end reads survived after trimming in all samples. The reads were then mapped using the “new tuxedo” package HISAT2 (41) to GRCh38 (hg38) genome. Annotation of properly oriented paired reads to exons was done using FeatureCounts v. 2.0.1 (42) using these additional parameters: –s 2 –t exon –g gene_id. The datasets presented in this study can be found in the GEO repository with accession number, GSE168642.

### Differential Gene Expression Analysis

Differential gene expression analysis of count tables obtained from FeatureCounts (n = 3, for each treatment) was performed using DESeq2 v.1.26.0 (43). To normalize the compositional variation in samples’ libraries, we utilized the median of ratios method of the DESeq2 package. To obtain biologically relevant genes, for most analyses, we kept the differential gene expression (DGE) cutoff as logFC >1.5 or <1.5 and FDR <0.05. Enrichment of biological processes was performed using Gene Set Enrichment

Analysis (http://software.broadinstitute.org/gsea) with GO terms was obtained from MSigDB. For most significant pathway enrichment and comparative pathways across samples, we used ReactomePA v.1.30.0 package of R (44). Statistics was performed using Benjamini–Hochberg method with p < 0.01.

Protein–protein interaction maps were generated from the significant gene lists between treatments using Cytoscape v.3.8.0 (45) and Metascape (46). Two-dimensional cluster mapping of pathways was performed *via* ViSEAGO v.1.1.0 (47) package of R. Factor enrichment from DGE analysis was performed using EnrichR web server tool (48).

### Immunostaining

For cell surface staining, CD3-BV786 (clone SK7), γδ TCR-APC (clone B1), Vδ2 TCR-PE (clone B6), and CCR4-PE (clone 1G1) were obtained from BD Biosciences. Vγ9Vδ2 T cell cultures were stimulated with interleukin 2 (IL2), HDMAPP + IL2, IPP + IL2 or anti-CD3 + IL2 for 72 h as described above. After 72 h, Vγ9Vδ2 T cells were washed with PBS and cold fixed with 1% paraformaldehyde for 15 min at 4°C to assess the membrane expression of CCR4. Intracellular staining of IL17A and IL17F was performed using IL17A-AF647 (clone BL168, BD Biosciences) and IL17FPerCP eFluor 710 (clone SHLR17, Invitrogen) antibodies respectively. After 72 h culture, Vγ9Vδ2 T cells were washed with 1× PBS and restimulated with phorbol 12-myristate 13-acetate (PMA; 50 ng/ml, Sigma-Aldrich) and ionomycin (1 μg/ml, Sigma-Aldrich) for 4 h in the presence of Brefeldin A (5 μg/ml, Sigma-Aldrich). Vγ9Vδ2 T cells were washed with 1× PBS and cold fixed with 1% paraformaldehyde for 15 min at 4°C, followed by permeabilization with 0.1% saponin for 5 min at room temperature. Cells were washed and stained with respective antibodies for 30 min at 4°C. A minimum of 50,000 events were acquired using FACS Aria III flow cytometer and analysis was carried out using FlowJo software (TreeStar, Ashland, OR, USA).

### Cytometric Bead Array

Cell-free supernatants were collected from Vγ9Vδ2 T cultures after 72 h of stimulation with IL2, HDMAPP + IL2, IPP + IL2, or anti-CD3 + IL2. Secreted cytokines interferon-γ (IFN-γ), IL17A, IL4, IL6, IL10, and TNF in the culture supernatants were measured using human Th1/Th2/Th17 cytokine cytometric bead array (CBA) kit as per manufacturer’s instructions (BD Biosciences, USA). Samples were acquired on FACS Aria I and analyzed using BD FCAP Array (BD Biosciences, USA). Statistical analysis was done by Student’s t-test using GraphPad Prism software (GraphPad Software Inc., CA, USA).

### cDNA Synthesis and Quantitative Real-Time PCR

Total RNA was isolated after each treatment using TriZol reagent (Invitrogen). RNA was quantitated using Nanodrop (Thermo), and ~500 ng RNA was processed for complementary DNA (cDNA) synthesis. Following DNase I (Roche) digestion, RNA was subjected to cDNA synthesis using iScript cDNA synthesis kit (BioRad). Quantitative RT-PCR analyses were performed using SYBR green qPCR master mix (Takara) at the following PCR conditions: step 1, 95°C, 5 min; step 2, 95°C, 30 s, 60°C, 30 s, and 72°C, 30 s for 40 cycles. The change in gene expression was calculated using the formula ΔCt = Ct Target − Ct Control. Normalized transcript expression was calculated using the equation 2 − (ΔCt), where ΔCt = Ct Target − Ct Control. The oligonucleotide primer sequences used for quantitative real-time PCR (qRT-PCR) analyses are listed in the **Supplementary Table S1**.

## Results

**Figure 1:**
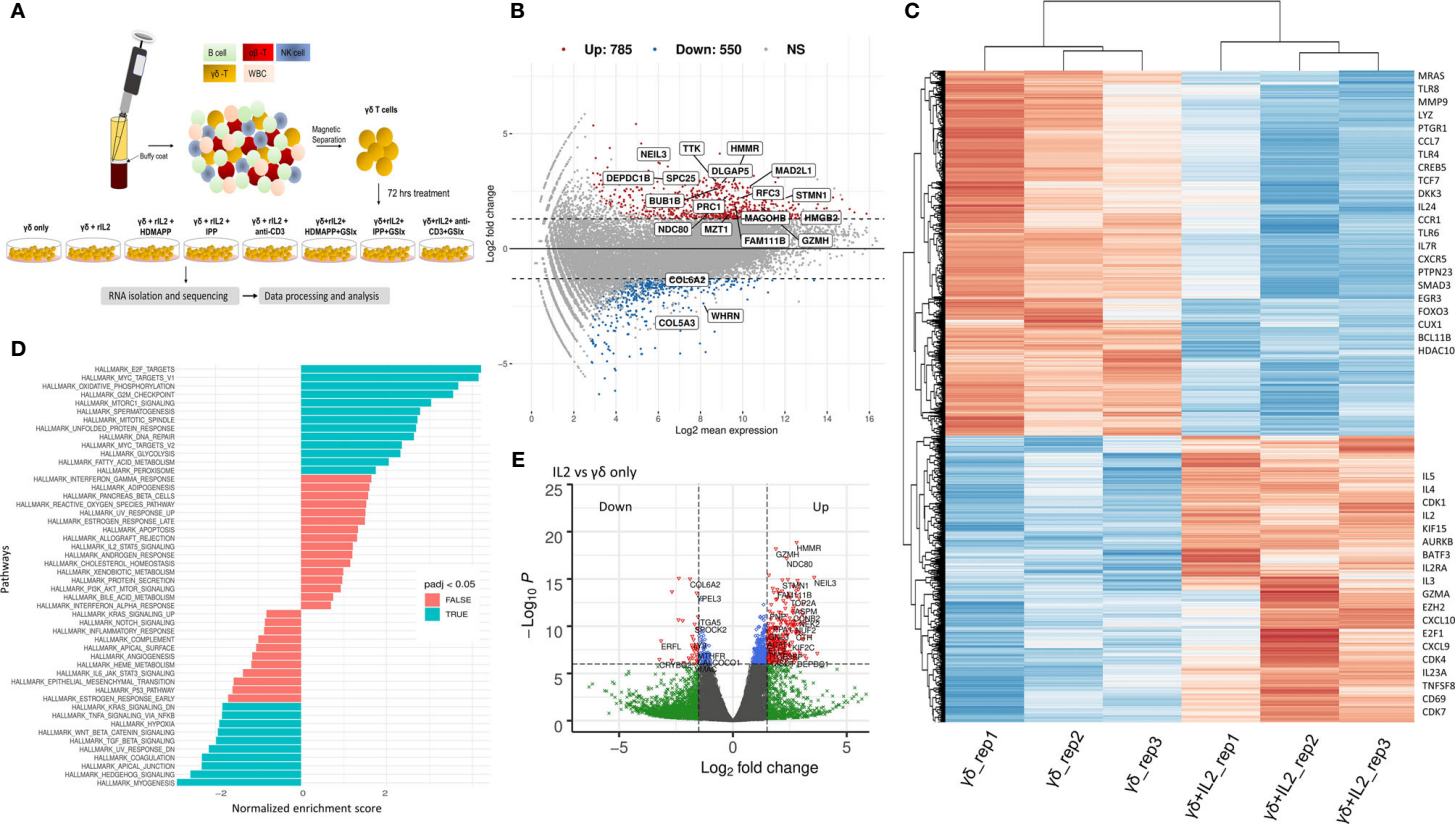
IL2 mediated signaling reshapes γδ T cell gene expression. **(A)** Schematic of γδ isolation, treatment regime, and RNA-seq. **(B)** Overall gene dysregulation upon IL2 treatment was plotted using MA function for fold change *vs.* normalized expression (n = 3). Boxed genes shown are dysregulated by 2.5-logFC with an FDR of 0.05. **(C)** Clustered heatmap for topmost differentially regulated genes upon IL2 treatment (n = 3 for each treatment). Key differentially regulated cytokine and effector genes are labeled. **(D)** Functional GeneSet Enrichment Analysis (fGSEA) was performed for Gene Ontology (GO) term or Hallmark enrichment terms. **(E)** Volcano plot for fold change *vs.* p-value. Intersecting lines indicate the logFC cutoff. Colors as indicated; gray, NS; blue, logP = −6; green, FC = 1.5; red, FC = 1.5 and logP = −6.

To dissect the role of signaling pathways in the tumor microenvironment involved in the functioning/survival of Vγ9Vδ2 T cells, we employed *ex vivo* culture of Vγ9Vδ2 T cells followed by high-throughput transcriptome analysis. Briefly, Vγ9Vδ2 T cells were isolated from peripheral blood of healthy donors using magnetic sorting with >95% purity (**Supplementary Figure S1**). Vγ9Vδ2 T cells (1 × 10^6^ per well) were treated with different combinations of stimulants in three replicates as depicted in the experimental workflow in **Figure 1A**. After 72 h of incubation, total RNA was isolated and subjected to transcriptome analysis using RNA-seq. Sequencing reads for all individual samples ranged from 40 to 70M (**Supplementary Table S2**). Postalignment, more than 93% coverage was obtained for each adapter-trimmed sequencing data. Although we found a moderate ~10%–30% multimapped reads, we only considered uniquely mapped reads for all further analysis. For analysis of the differentially expressed (DE) genes, we have utilized the pipeline for known-gene assembly (GRCh38)—summarization tools like Feature Counts followed by Deseq2 and downstream packages in the R environment were used.

### IL2-Mediated Signaling Reshapes Vγ9Vδ2 T Cell Gene-Expression Profile

Signaling *via* cytokine IL2 has been implicated in the survival and effector and memory functions of CD4^+^ and CD8^+^ αβ T cells (49). It has been shown that IL2 is important for γδ T-cell survival and proliferation (50). Additionally, γδ T cells produce IFN-γ in the presence of IL2. This occurs *via de novo* induction of T-bet and Eomesodermin (Eomes) by IL2 (51). We, therefore, wished to dissect the global transcriptional changes that occur in Vγ9Vδ2 T cells upon IL2 treatment. We performed DGE analysis of IL2-treated *vs.* untreated Vγ9Vδ2 T cells and mostly focused on only protein-coding transcripts with 38,557 genes annotated from the reference assembly. As shown *via* MA plot, there were more than 1,300 genes differentially regulated upon IL2 treatment with a stringent cutoff of >2.5 (logFC) (**Figure 1B**), although, overall, 4,743 genes have been significantly dysregulated (**Supplementary Table S3**). We observed that about 71% of these genes were upregulated, suggesting that IL2 signal leads to better survival and effector function potentially *via* inducing expression of different signaling pathways. Furthermore, we performed hierarchical clustering for top 500 DE genes of both the datasets (**Figure 1C**, also see **Supplementary Figure S2**). We observed that many genes among replicates do not show coherent expression. This could be due to varying states of IL2 signaling in the isolated pan γδ T cells from different donors. As shown, we found cell-cycle genes, such as CDK1, CDK7, and AURKB, and cytokine genes, such as IL2RA, IL2, TNFSF8, and IL5, among others, were upregulated, which is in line with the previously reported effect of IL2 on cultured Vγ9Vδ2 T cells (52). The top downregulated genes consist of ligands such as CCL7 and CXCL16 (**Supplementary Table S3**). We also performed gene-set enrichment analysis (GSEA) of DE genes between the two conditions using the Gene Ontology (GO) term database. We observed that 91 GO terms were enriched using p < 10^−5^ and plotted both positively and negatively regulated processes with false discovery rate (FDR) cutoff as 0.05 (**Figure 1D**). Here, among the most significantly enriched processes were E2F-mediated transcription, cell cycle, MTORC1 signaling, and Wnt–β-catenin signaling axis. Furthermore, multiple direct targets of Wnt/β-catenin signaling including DKK3, MMP9, and TCF7 were dysregulated. As expected, a large number of genes were differentially regulated in the presence of IL2, even with higher cutoffs for both p-values and logFC as depicted by the volcano plot (**Figure 1E**). From these observations, we determined the overall gene expression dynamics of Vγ9Vδ2 T cells upon IL2 signaling and the pathways that get most affected.

### Transcriptional Dynamics Upon Phosphoantigen-Driven Activation of Vγ9Vδ2 T Cells

**Figure 2:**
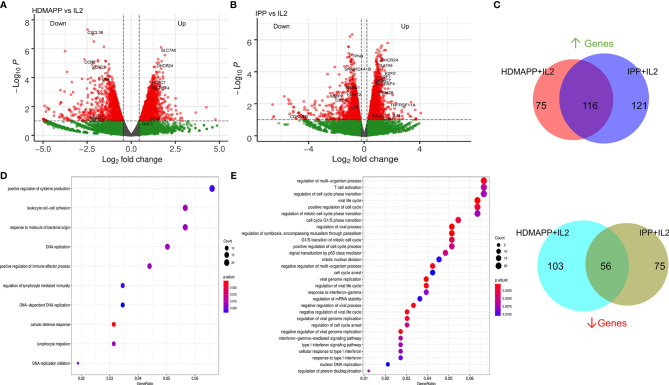
Transcriptional dynamics upon phosphoantigen-driven activation of γδ T cells. **(A)** Differential gene expression of HDMAPP + IL2 *vs.* IL2 treatment was performed showing key labels above a selected cutoff range. (n = 3). **(B)** Volcano plot for IPP + IL2 treatment compared for DGE over IL2 (n = 3). **(C)** Differentially regulated genes for HDMAPP + IL2 over IL2 and IPP + IL2 over IL2 with FDR < 0.05 were segregated in up- and downregulated genes for each treatment pair. Venn diagrams were plotted for common up- and downmodulated genes comparing both the phosphoantigen conditions. **(D, E)** Enrichment dot plots for HDMAPP + IL2 over IL2 and IPP + IL2 over IL2 treatments, showing most significantly enriched GO terms.

After determining how IL2-mediated responses of Vγ9Vδ2 T cells translate into transcriptional changes, we wanted to understand how non-peptide phoshoantigens IPP and HDMAPP affect the transcriptional program of Vγ9Vδ2 T cells. Our previous data demonstrated that treatment of γδ T cells with either IPP or a similar bacterial metabolite intermediate HDMAPP resulted in activation and increased cytotoxic potential *in vitro* (28, 53). We also showed that T-bet, Eomes, and IFN-γ display a significant degree of upregulation postactivation (23, 28). To understand the effect of phosphoantigen stimulation, we compared RNA-seq data of HDMAPP + IL2-treated Vγ9Vδ2 T cells to only IL2-treated cells as shown in **Figure 2A**. We observed an increase in the transcripts of proinflammatory cytokine receptor for TNF-α (TNFRS4) and AP1 family proteins FOS and JUN in HDMAPP + IL2-treated Vγ9Vδ2 T cells. Surprisingly, we observed only a mild but statistically insignificant (at p < 0.001) increase in IFN-γ levels. Similarly, we analyzed IPP + IL2 *vs.* IL2-only datasets and observed that expression of IL13 and CCR4 increased more than 2-fold (**Figure 2B**). We also observed an increase in the SLC family protein SLC7A5, an amino acid transporter involved in TCR activation of CD4 and CD8 T cells (54, 55), in both the phosphoantigen treatments over IL2 only dataset. We also validated the protein expression of IFN-γ, IL17F along with IL17A, and CCR4 receptor upon phosphoantigen or anti-CD3 stimulation of Vγ9Vδ2 T cells using CBA and flow cytometry, which has been discussed below. Although IL13 is known to be expressed exclusively by skin-residing γδ T cells displaying Vγ5Vδ1 TCR in mice model (56), phosphoantigen-treated human Vδ2 γδ T cells also secrete IL13 along with other cytokines (57). Since IPP and HDMAPP are similar metabolic intermediates in the presence of which γδ T cells seem to function similarly *in vitro* (53), we expected to observe overlapping extent of differential gene expression that these two treatments might confer when compared to IL2-only samples. As seen in **Figure 2C**, 61% and 50% overlap was observed for commonly upregulated genes in HDMAPP + IL2 *vs.* IL2 and IPP + IL2 *vs.* IL2 datasets, respectively. In contrast, we observed 35% and 44% overlap between commonly downregulated genes among the two treatments. Given that many of the genes differentially regulated are not the same in both up- and downregulated Venn sets in the two mentioned conditions, we wished to monitor which pathways are most significantly enriched for both HDMAPP and IPP treatments. In **Figures 2D, E**, we performed pathway analysis using enrich GO terms. We observed that most of the pathways with FDR <0.006 are different in both treatments, with lymphocyte-specific pathways such as cytokine production and lymphocyte migration enriched in the HDMAPP treatment. The cell-cycle and interferon-response pathways were most abundant in the IPP-treated Vγ9Vδ2 T cells. These results indicated that HDMAPP treatment can induce a robust immune response *via* activation of T-cell-specific factors. On the other hand, IPP treatment seems to be more important in cell expansion and immune response.

### Distinct and Common Transcriptional Pathways Activated in Vγ9Vδ2 T Cells Upon Stimulation With Phosphoantigens

**Figure 3:**
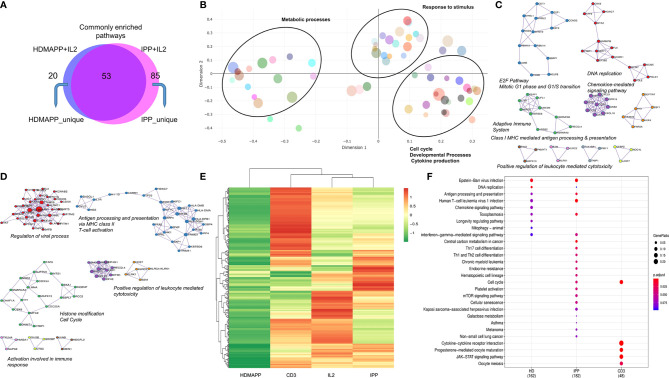
Distinct transcriptional pathways are activated *via* each phosphoantigen. **(A)** Intersection of significant GO terms enriched for HDMAPP and IPP treatments. **(B)** A clustering-based semantic similarity mapping of most significant GO terms, showing how various pathways activated in HDMAPP treatment are distanced in a multidimensional scale. **(C, D)** Protein–protein interaction map for genes differentially enriched upon HDMAPP + IL2 and IPP + IL2 treatments over IL2, respectively. Most enriched corresponding processes are labeled. The maps were generated using Cytoscape with BioGrid, InWeb_IM, and OmniPath databases. **(E)** Clustered heatmap of most differentially regulated genes with each mentioned stimulation (also see **Supplementary Figure S3**). **(F)** KEGG pathway comparison was done for HDMAPP, IPP, and anti-CD3 stimulations. The dots indicate both significant enrichment and p-value for the mentioned pathway. n = 3 was used for each analysis.

Based on the GO-term analysis (**Figures 2D, E**), we next studied the significantly enriched pathways in the two activation treatments. We performed Reactome pathway analysis to link the related pathways *via* network and plotted Venn diagram for common and unique pathways between HDMAPP and IPP stimulations (**Figure 3A**). We furthermore performed a distance mapping of clustered GO terms in which we first clustered related GO terms followed by computing their distance *via* semantic similarity with respect to each other on the XY axes. As shown in **Figure 3B**, most abundant clusters in HDMAPP treatment are of metabolic, stimuli responses and immune-related processes. Of note, clusters of T-cell activation and cell cycle have many overlapping GO terms, corroborating that these two “umbrella” processes have mutual crosstalk in Vγ9Vδ2 T cells, which also supports the previously reported microarray-based gene expression profiling upon phosphoantigen BrHPP treatment (58). Interestingly, since we probed for the effects of only phosphoantigen treatment (HDMAPP and IPP) autonomous to IL2 influence, we observed pathways enriched only as a result of phosphoantigen treatments such as T-cell activation and leukocyte differentiation. We furthermore probed into the TF and cytokine crosstalk *via* protein–protein interaction in the two treatments. Upon performing network analysis of interacting proteins with a threshold of >5 GO terms, many TFs and cytokines were enriched in HDMAPP (**Figure 3C**) and IPP (**Figure 3D**) DGEs over IL2 only datasets. Despite processes including “chemokine signaling,” “leukocyte-mediated cytotoxicity” and “antigen presentation” being enriched in both conditions, they share only few TFs and chemokine receptors. It is also known that activation through anti-CD3 leads to γδ T-cell activation and enhanced effector response (28), although there is a non-redundant requirement of anti-CD28 signal as well (50). But since CD28 signaling activates IL2-mediated pathways (50), we sought to compare dataset of anti-CD3+ IL2-treated Vγ9Vδ2 T cells with other treatments. Hierarchical clustering of all four treatments—IL2 only, HDMAPP + IL2, IPP + IL2, and anti-CD3+ IL2 was performed. We observed four major clusters based on DGE across conditions (**Figure 3E** and **Supplementary Figure S3**). It is apparent that numerous top upregulated genes in anti-CD3 condition are not induced in IPP and HDMAPP-treated Vγ9Vδ2 T cells (also see **Supplementary Tables S4–S6**). We further performed the Kyoto Encyclopedia of Genes and Genomes (KEGG) pathway comparison for the three activation treatments with FDR <0.05 (**Figure 3F**). Following the DGE profiles as shown in **Figure 3E** and **Supplementary Figure S4**, we observed that most significantly enriched pathways have only a few common elements such as “cell cycle” and “cytokine –chemokine signaling”. In terms of effector function, since majority of the γδ T cells cultured were of Vγ9Vδ2 type (**Supplementary Figure S1**) and produce IFN-γ upon phosphoantigen treatment (1, 53), both IPP and HDMAPP treatments showed enriched IFN signaling. Collectively, these results show that both common and distinct metabolic and activation pathways are induced in γδ T cells upon phosphoantigen/anti-CD3 antibody treatment.

### Inhibition of Notch Signaling Disrupts Effector Signaling of Vγ9Vδ2 T Cells

**Figure 4:**
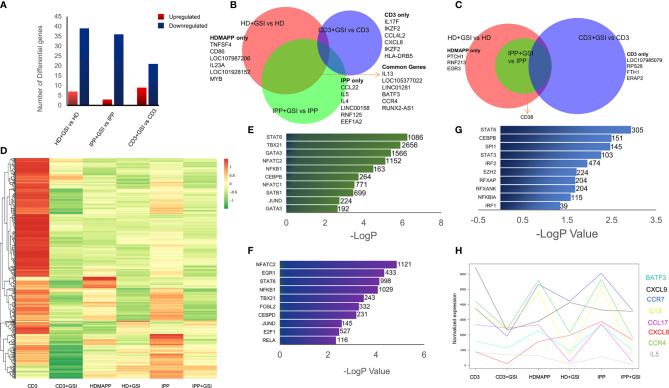
Inhibition of Notch signaling disrupts effector signaling of γδ T cells. **(A)** Total number of up- and downregulated genes in each activation *vs* corresponding activation + Notch-inhibition condition. DGE was performed with Wald’s test, logFC > 0.5 and FDR < 0.05. **(B, C)** Common genes down- and upregulated between each Notch inhibited dataset. Some of the key factors dysregulated are shown for both overlapping and individual treatments. **(D)** Heatmap showing differential clustering of γδ T-cell-activating treatments and their combination with Notch inhibition (n = 3). **(E–G)** Enrichr analysis was performed on each DGE analyzed pair—IPP + GSI *vs.* IPP, HDMAPP + GSI *vs.* HD, and anti-CD3 + GSI *vs.* CD3, respectively. The most significantly affected TFs-mediated pathways are shown, ordered according to their −logP values. Combined Enrichr score is indicated for each factor as shown. **(H)** Normalized gene expression profiles for key cytokines across different activations and activations coupled with Notch inhibition (n = 3). Statistics was performed using the likelihood ratio test, with FDR < 0.05.

Notch signaling plays an essential role in T-cell activation and differentiation (25), and their antitumor potential (27). Our previously published data established that phosphoantigen or anti-CD3-driven *in vitro* activation of γδ T cells leads to induction of Notch signaling by directly regulating Notch expression (28), and blockade of Notch pathway *via* gamma secretase inhibitor (GSI-X) or siRNA results in decreased expression of effector molecules and hampered cytotoxic activity against tumor cell lines. To evaluate the global transcriptional phenomenon upon Notch inhibition in activated Vγ9Vδ2 T cells, we treated Vγ9Vδ2 T cells with a combination of either IPP + IL2 + GSI, HDMAPP + IL2 + GSI, or anti-CD3 + IL2 + GSI and carried out RNA-seq. The datasets obtained were compared with their respective activated-only counterparts for DGE analysis. Notch inhibition resulted in a greater number of downregulated genes compared to positive regulation for both phosphoantigens (cutoff >0.5logFC, FDR <0.05) (**Figure 4A** and **Supplementary Figure S4)**, and in the case of anti-CD3 + IL2 + GSI *vs.* anti-CD3 + IL2 treatments. As Notch signaling is known to feedback upon TCR signaling positively, we observed that inhibition of Notch signaling leads to downregulation of TCR-specific TFs such as BATF and RUNX2. and IL13 and CCR4 among cytokines and cytokine receptors (**Figure 4B**), while the top positively regulated genes included EGR3 and CD38 (**Figure 4C**). Although both BATF1 and 3 were downregulated commonly in all three inhibitory conditions, only BATF3 fulfilled our cutoff criterion. DGE analysis was also performed across activating and inhibitory conditions using the likelihood ratio test (similar to ANOVA) as shown *via* clustered heatmap (**Figure 4D**, also see **Supplementary Tables S7–S9** for significant DGs). Furthermore, we also investigated which molecular pathways are most affected under Notch inhibition. We performed enrichment analysis using Enrichr (48) for IPP + GSI *vs.* IPP DE genes (**Figure 4E** and **Supplementary Figure S5**) and observed that the most affected pathways involve STAT6, T-BET, and GATA3-mediated signaling. Strikingly, signaling *via* SATB1, an early TCR-responsive chromatin organizer (59), is also affected by Notch inhibition. In a similar analysis for HDMAPP + GSI *vs.* HDMAPP dataset, we found that highest Enrichr scoring factors were STAT family proteins and NFATC2, both implicated in immune responses (**Figure 4F** and **Supplementary Figure S5**). Interestingly, for anti-CD3 + GSI, we observed most dramatic changes in chromatin-related factors including CEBP and EZH2 along with the STAT family proteins STAT6 and STAT3 (**Figure 4G**). Since cytokine signaling is important in the effector function of Vγ9Vδ2 T cells, we also plotted expression profiles of most DE cytokine and/or cytokine/chemokine receptor genes to represent the effect of Notch inhibition on activation of inflammatory response (**Figure 4H**). We observed an antagonistic effect of Notch inhibition on activation-induced genes such as CCR4, IL5, IL13, and CXCL8. These results collectively point out that there are distinct TF networks enriched upon different activation treatments, thus modulating the effector function of Vγ9Vδ2 T cells uniquely. Our results also indicate that inhibition of Notch signaling disrupts the phosphoantigen-mediated effector gene signature of Vγ9Vδ2 T cells hampering their function (28).

### Validation of Transcriptomics Data

**Figure 5:**
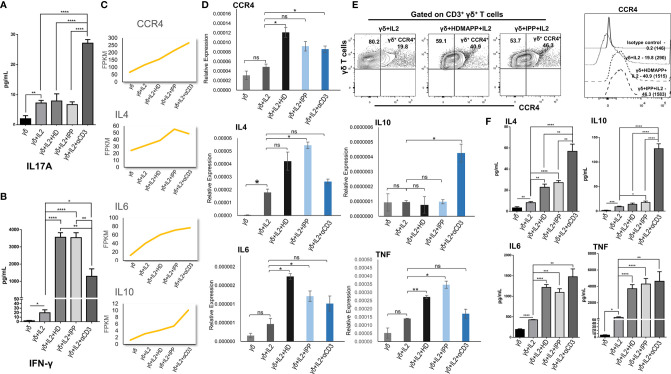
Validation of transcriptomics data. The expression of cytokines, surface receptors, and key genes in differentially stimulated γδ T cells isolated from peripheral blood lymphocytes of healthy individuals (n = 3) were assessed by cytokine bead array (CBA), multicolor flow cytometry, and quantitative real-time PCR (qRT-PCR), respectively. **(A, B)** The cell-free supernatants collected after 72 h were assessed for secreted cytokines IL17A and interferon-γ (IFN-γ) using human Th1/Th2/Th17 cytokine CBA kit. **(C)** Normalized FPKM plots showing the expression change of selected targets upon IL2, HDMAPP, IPP, and anti-CD3 treatments of γδ T cells. **(D)** qRT-PCR analyses of the selected targets as shown in panel **(C, E)** Representative figure showing the expression of CCR4 gated on CD3+ γδ+ T cells in IL2, HDMAPP + IL2, or IPP + IL2 stimulated γδ T cells after 72 h culture. The MFI of CCR4 expression on differentially stimulated γδ T cells was analyzed after 72 h culture. The values in the inset represent the percent positive population, and the values in brackets represent the MFI of CCR4 expression. **(F)** CBA was used to ascertain the secreted protein levels of the target cytokines after 72 h culture as tested in panel **(D)** All the results indicated are mean ± standard error of the mean (SEM) of three independent experiments (*p < 0.05; **p < 0.01; ***p < 0.001; ****p < 0.0001, ns, non-significant).

We validated some of the prominent transcriptomic signatures obtained in Vγ9Vδ2 T cells as mentioned previously upon stimulation with phosphoantigens (HDMAPP and IPP) or anti-CD3 by analyzing the expression of specific cytokines and surface receptors. The intracellular expression of IL17A and IL17F and the secreted cytokines (IL17A, IFN-γ, IL4, IL6, IL10, and TNF) in the cell-free culture supernatants in stimulated Vγ9Vδ2 T cells was analyzed. Higher levels of IL17A were secreted by Vγ9Vδ2 T cells upon anti-CD3 stimulation as compared to phosphoantigen (IPP and HDMAPP) and only IL2 stimulation (**Figure 5A**), which corroborates our transcript data (**Supplementary Figure S6**). The intracellular expression of IL17A and IL17F in the anti-CD3-stimulated *vs.* phosphoantigen (HDMAPP and IPP) or IL2-stimulated Vγ9Vδ2 T cells also supported this observation (**Supplementary Figure S6**). Although transcriptomics data showed insignificant change in expression of IFN-γ on phosphoantigen and anti-CD3 stimulation as compared to only IL2 stimulation, significant differences were noted at the protein level. On the contrary, we observed that Vγ9Vδ2 T cells upon phosphoantigen and anti-CD3 stimulation secreted higher levels of IFN-γ (**Figure 5B**) as compared to only IL2. Interestingly, activation of Vγ9Vδ2 T cells with phosphoantigens led to more robust increase in secreted levels of IFN-γ in comparison with anti-CD3 stimulation (**Figure 5B**), which can be corroborated with the KEGG pathway comparison made at the mRNA level (**Figure 3F**). We further validated the expression of many of the cytokines from our expression analysis as shown in **Figure 5C** at the mRNA level (**Figure 5D**) relative to 18s rRNA transcripts in γδ, γδ + IL2, γδ + IL2 + HDMAPP, γδ + IL2 + IPP, and γδ + IL2 + anti-CD3 conditions. We observed coherent gene expression profiles of cytokines such as IL4, IL6, and IL10 and cytokine receptor CCR4 to the fragments per kilobase of transcript per million mapped reads (FPKM) data plotted for each of the tested genes (**Figure 5C**). We furthermore examined the expression of surface receptor CCR4 on stimulated Vγ9Vδ2 T cells, since it was upregulated on phosphoantigen (HDMAPP and IPP) stimulations at the mRNA level (**Figures 2A, B**). We found similar upregulation of CCR4 surface expression on both HDMAPP- and IPP-stimulated Vγ9Vδ2 T cells (**Figure 5E**). Additionally, we probed the levels of secreted cytokines validated *via* qRT-PCR to monitor the effect on the protein expression (**Figure 5F**) and observed that both phosphoantigen and anti-CD3 treatments induce the expression of these cytokine genes over IL2-only samples. We also validated the transcript expression of many TFs induced by phosphoantigen/anti-CD3 treatments and downregulated upon Notch inhibition in our analysis (**Supplementary Figure S7**). Collectively, these results corroborated the results of the transcriptome analysis.

## Discussion

The present study reveals the transcriptional dynamics during γδ T cell activation. We showed that stimulations of TCR using phosphoantigen (IPP and HDMAPP) and anti-CD3 antibody treatments *in vitro* can lead to the induction of both distinct and similar transcriptional programs in Vγ9Vδ2 T cells, although the effector molecules such as IFN-γ, Perforin-1 (PRF1), and tumor necrosis factor receptor superfamily (TNFRSF) members are positively regulated across various activation methods. It has been reported that HDMAPP is 1,000-fold more potent than IPP in activating Vγ9Vδ2 T cells. Binding studies using isothermal titration calorimetry have demonstrated that HDMAPP binds to B30.2 intracellular domain of BTN3A1 with an affinity of 0.5 µM, whereas IPP bound with 0.5 mM affinity reflecting the potency differences between these two agonists in activating γδ T cells (20). HMBPP stimulation of γδ T cells induces formation of high-density nanoclusters of Vγ9Vδ2 TCR on the membranes of these cells and maintain high TCR surface expression in contrast to extensive internalization of the TCR observed after anti-CD3 stimulation (60–62). There are fundamental differences in activation of γδ T cells by anti-CD3 and phosphoantigens. Phosphoantigens do not trigger the CD3 conformational change in γδ TCR (63). Anti-CD3 activation enhances the recruitment of non-catalytic region of tyrosine kinase adapter protein 1 (NCK) to the γδ TCR, which helps in stabilizing the receptor in an active conformation (64). We have also monitored expression of early activation markers, CD69 and CD25, on Vγ9Vδ2 T cells after stimulation with varying concentrations of HDMAPP and IPP. Our data showed that the differences we observed in transcriptomics data upon stimulation with these two phosphoantigens were not related to the differences in their activation status, as the levels of CD69 and CD25 were comparable at concentrations of HDMAPP (1 nM) and IPP (40 µM) used to activate the Vγ9Vδ2 T cells (**Supplementary Figure S8**). The differential intracellular protein expression of IL17A and IL17F in Vγ9Vδ2 T cells (**Supplementary Figure S6**) observed by us further corroborated the relatively reduced expression of IL17A compared to IL17F observed in our RNA-seq datasets. The Vγ9Vδ2 T cells stimulated with anti-CD3 secreted higher levels of IL17A compared to phosphoantigen (IPP and HDMAPP) stimulation (**Figure 5A** and **Supplementary Figure S6**). The results corroborated our earlier data wherein the IL17A-secreting γδ T cells were significantly elevated upon anti-CD3 stimulation in the presence of IL2 but not with HDMAPP, which also correlated with the expression of TF RORγT (62). Interestingly, Notch signaling has been reported to be involved in the activation of Th17 γδ T cells as well. IL17 and RORγT are direct transcriptional targets of Notch signaling (65), and Notch–Hes1 pathway has been shown to be involved in the murine intrathymic development of Th17 γδ T cells (66, 67). It is known that neonatal Vγ9Vδ2 T cells, under the influence of dendritic-cell-derived IL23 and TCR signaling, polarize to distinct subpopulations producing IFN-γ and IL17 (68). On the contrary, *in vitro* stimulation of adult Vγ9Vδ2 T cells, in the presence of IL23, produced IL17 in a minority of subjects compared to that observed in cord blood. The study shows that adult Vγ9Vδ2 T cells are biased towards IFN-γ production compared to their neonatal counterparts upon activation in the presence of IL23. It was therefore suggested that IL17-producing γδ T cells play a role against early life infections. Similar to our observation, overexpression of CCR4 receptor has been reported on γδ T cells isolated from peripheral blood of healthy individuals upon stimulation with IPP (69). Elevated expression of CCR4 receptor on human Vδ1 (70) and Vδ2 subsets (71) has also been reported under certain pathological conditions. The increase in the CCR4 levels on activated γδ T cells has been attributed to the migratory ability of these cells.

Recently, single-cell transcriptomic analysis revealed the hallmarks of distinction and overlap between different γδ T cells isolated from peripheral blood (36). Antigen stimulation in the periphery leads to specific expansion of Vγ9Vδ2 T cells throughout the postnatal life (72). Thus, this subset of γδ T cells has been at the center stage of γδ T-cell-based therapies (7). We showed that distinct transcription factors/pathways are activated contingent on the kind of antigen. To our surprise, IFN signaling was commonly enriched upon IPP and HDMAPP stimulation but not with anti-CD3, although IFN-γ levels increased modestly over IL2 treatment. Earlier gene expression data on TCR-associated transcriptional signatures of cytokines support high IFN-γ and low IL17 expression in γδ T cells stimulated with HMBPP. In previous studies, microarray data for differential gene expression of HMB-PP + IL2 and “resting” γδ T cells identified many key genes involved in the proliferation and cytotoxic activity of Vγ9Vδ2 T cells (58, 60). Many of these genes including MAPK13, SDF4, MAZ, and LAG3 are enriched in our datasets as well. Silva-Santos and colleagues have demonstrated the importance of phosphoinositide-3-kinase (PI3K)/AKT and extracellular signal-regulated kinase (ERK)/mitogen-activated protein kinase (MAPK) signaling for Vγ9Vδ2 proliferation and cytokine release (60). Similarly, we found that, in our datasets, genes encoding for Akt (AKT1), p38 (MAPK13), and c-JUN N-terminal kinase (JNK) (MAPK8) are upregulated upon phosphoantigen and anti-CD3 treatments (**Supplementary Figure S9**), thus conforming with previous findings. Further experiments are warranted to evaluate the effect of factor-specific ablation such as basic leucine zipper ATF-like transcription factor 3 (BATF3) and nuclear factor kappa B (NF-kB) on Vγ9Vδ2 T-cell activation.

Previous studies by Chiplunkar et al. have demonstrated an obligatory role of Notch signaling on Vγ9Vδ2 T cell effector function (28, 53). It has been shown using microarray data that inhibition of Notch signaling resulted in metabolic stress and activation deficit in B-cell precursors (73), although no such global studies in Vγ9Vδ2 subsets are done to our knowledge. To predict the mechanism of Notch-mediated transcriptional dynamics in TCR-sensitized Vγ9Vδ2 T cells, we utilized a similar inhibitory approach combined with antigenic or antibody treatments. Our RNA-seq analysis not only corroborated that effector molecules such as IFN-γ and Granzyme B are downregulated upon Notch blockade but also provided a large set of potential targets to explore in the future studies. Many clinical studies have attempted to harness the antitumor activity of γδ T cells through isolation of Vγ9Vδ2 T cells, their *ex vivo* expansion and phosphoantigen activation, and adoptive transfer in patients (74–76). Although there were no side effects, the success in these clinical studies was not substantial. This is attributed to the deficits in the knowledge of Vγ9Vδ2 TCR interaction dynamics with the stimulating antigen and the functional outset of such interaction (77). Our global transcriptome analysis pertaining to such *in vitro* activation treatments displays the range of pathways and highlights the key molecules affected therein. The functional role of these pathways/TFs needs to be ascertained with respect to antitumor cytotoxicity of Vγ9Vδ2 T cells and requires future experimentation.

In conclusion, we demonstrated that effector functions of Vγ9Vδ2 T cells from peripheral blood ensue from many immunological and metabolic pathways, and these pathways are disrupted in consequence to Notch signaling inhibition. The specific role of treatment-dependent factors can be altered *via* CRISPR/Cas9 methodology followed by antigen stimulation, which might lead to an improvement in anticancer potential of peripheral Vγ9Vδ2 T cells.

## Data Availability Statement

The datasets presented in this study can be found in online repositories. The names of the repository/repositories and accession number(s) can be found below: NCBI GEO, GSE168642 (https://www.ncbi.nlm.nih.gov/geo/query/acc.cgi?acc=GSE168642).

## Author Contributions

AM analyzed the transcription data. AM, SAB, CSP, and SKS performed the experimental work and data analysis. AM, SG, and SC wrote the manuscript. SG and SC conceived and supervised the study. Funding was provided by SC and SG. All authors contributed to the article and approved the submitted version.

## Funding

This work was supported by a grant from the Unit of Excellence (BT/MED/30/SP11288/2015) program of the Department of Biotechnology (DBT), Government of India, to SC and SG. SG is a recipient of the JC Bose Fellowship (JCB/2019/000013) from the Science and Engineering Research Board, Government of India. AM is supported by a fellowship from the Council of Scientific and Industrial Research, Government of India. SB is supported by a fellowship from the Department of Biotechnology, Government of India. SKS is supported by fellowship from Department of Atomic Energy, Government of India.

## Conflict of Interest

The authors declare that the research was conducted in the absence of any commercial or financial relationships that could be construed as a potential conflict of interest.

## Publisher’s Note

All claims expressed in this article are solely those of the authors and do not necessarily represent those of their affiliated organizations, or those of the publisher, the editors and the reviewers. Any product that may be evaluated in this article, or claim that may be made by its manufacturer, is not guaranteed or endorsed by the publisher.
