# Supplementary material for: Transcriptome Signature of Vγ9Vδ2 T Cells Treated With Phosphoantigens and Notch Inhibitor Reveals Interplay Between TCR and Notch Signaling Pathways
Source: Front Immunol. 2021 Aug 30;12:660361. doi: 10.3389/fimmu.2021.660361 (PMC8435775; doi:10.3389/fimmu.2021.660361)
Supplement: Supplementary file 1 [file DataSheet_1.pdf]

Supplementary Figure 1

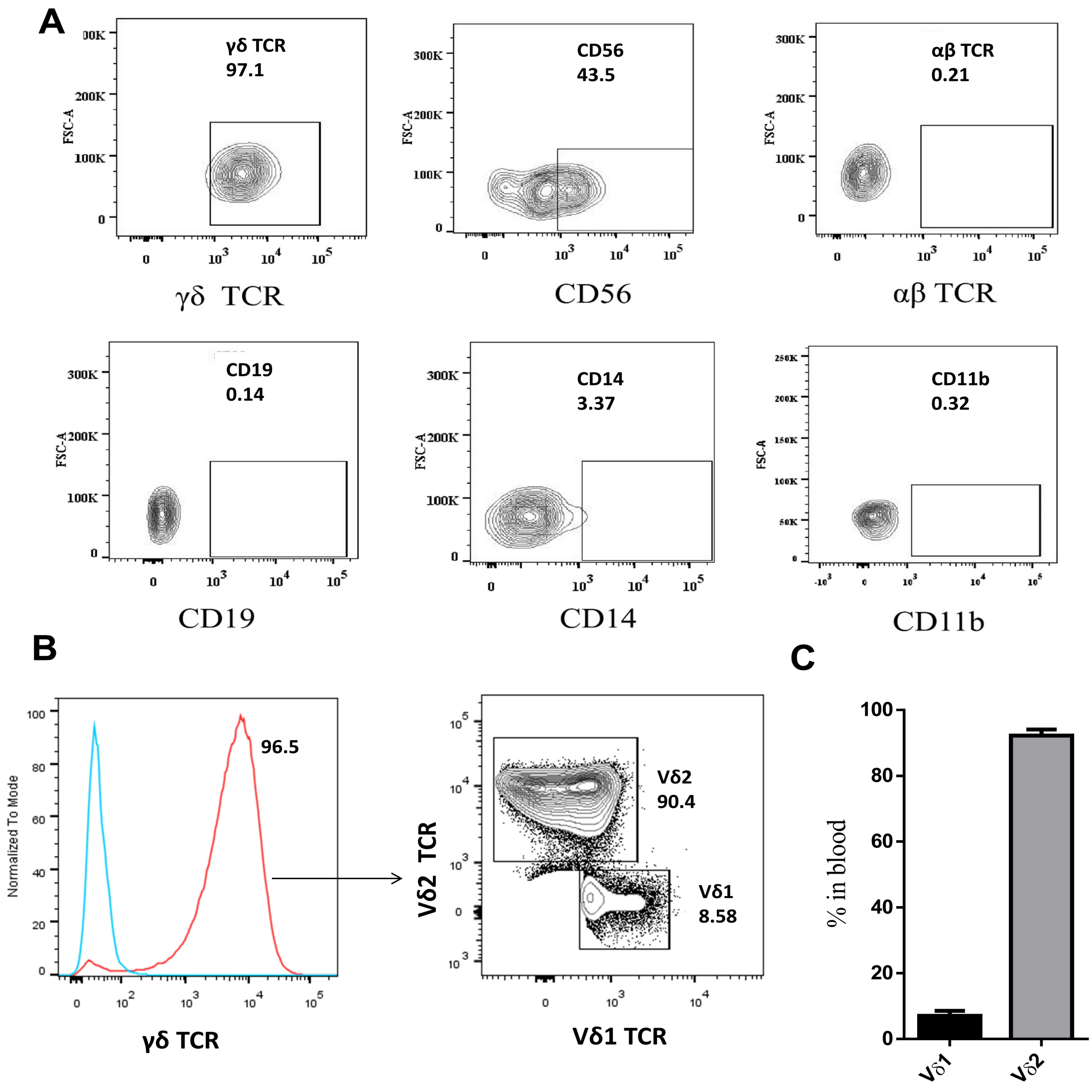

Supplementary Figure S1: Purity of isolated Vγ9Vδ2 T cells. Flow cytometry analysis of magnetically purified γδ T cells from fresh PBMCs (n=3). A. Representative figure showing expression of surface markers γδ TCR, αβ TCR, CD14, CD19, CD56 and CD11b on purified γδ T cells. Purity of γδ T cells was >95% in all 3 samples obtained from healthy individuals used for RNA-seq. B. Distribution of Vδ2 TCR and Vδ1 TCR subsets of γδ T cells in the purified γδ T cells isolated from PBMCs. C. Percent populations of Vδ1 and Vδ2 in peripheral blood. Data represent mean ± standard error of the mean (S.E.M.).

Supplementary Figure 2

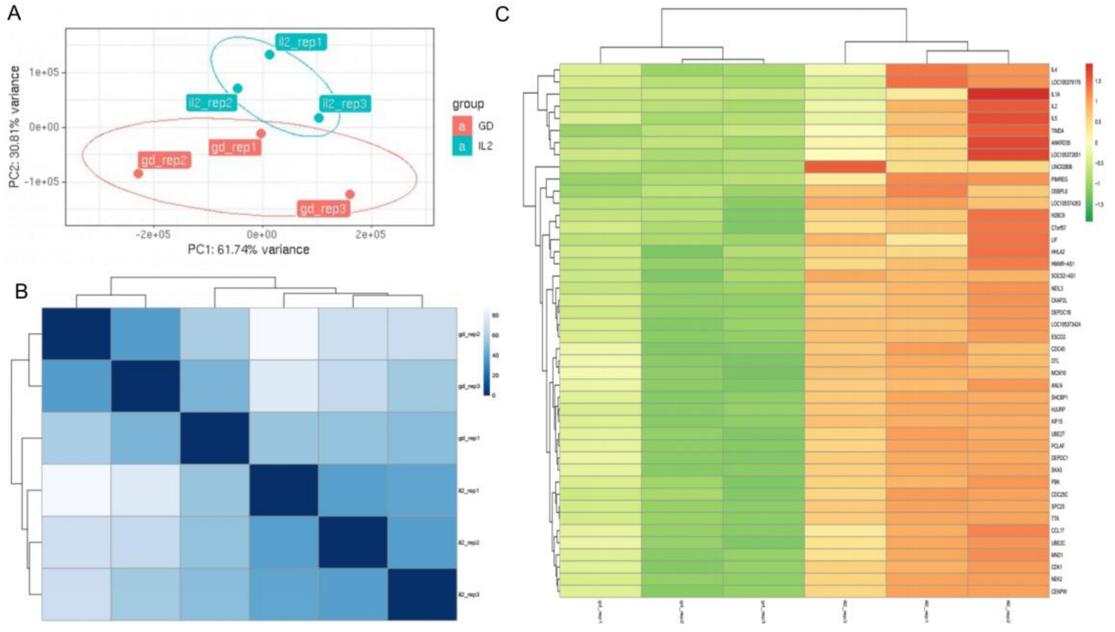

Supplementary Figure S2. IL2 modulates major gene expression in gamma-delta T cells. A. Principal component (PC) analysis showing the variance between the three replicates each of the Il2 treated vs the untreated gamma-delta transcriptomes (n=3000). B. Based on the PC analysis, distance-matrix analysis was performed to select the less variable replicates for downstream analysis. C. Heirarchical clustered-Heatmap to show the top 40 upregulated genes with FDR<0.01 and logFC>2.5.

Supplementary Figure 3

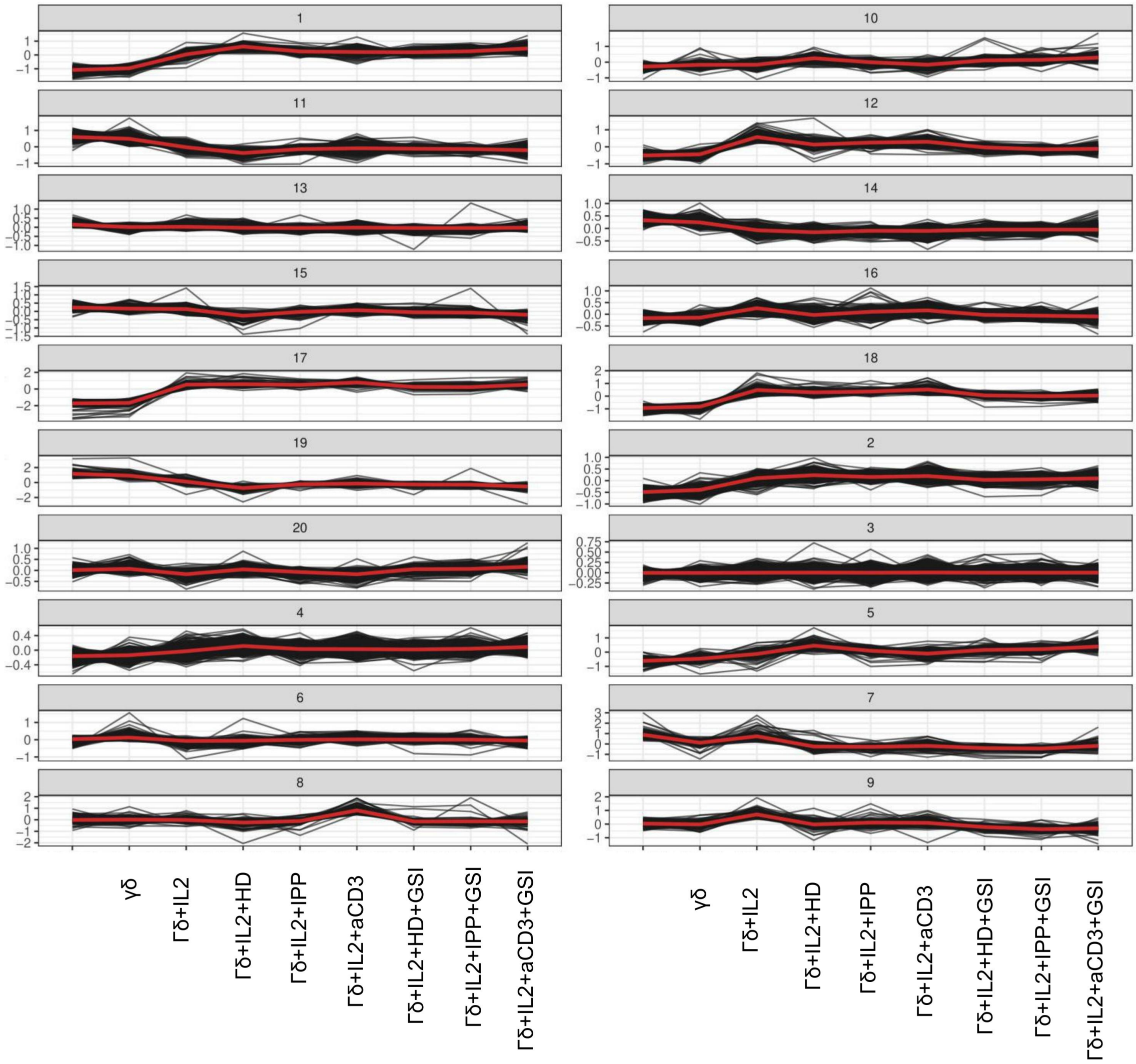

Supplementary Figure S3. k-means clustering of significantly differentially expressed genes. Normalized, log-transformed k-means clusters of significantly variable genes as determined by the Chi-Square test (FDR<0.05). Iterations=20.

Supplementary Figure 4

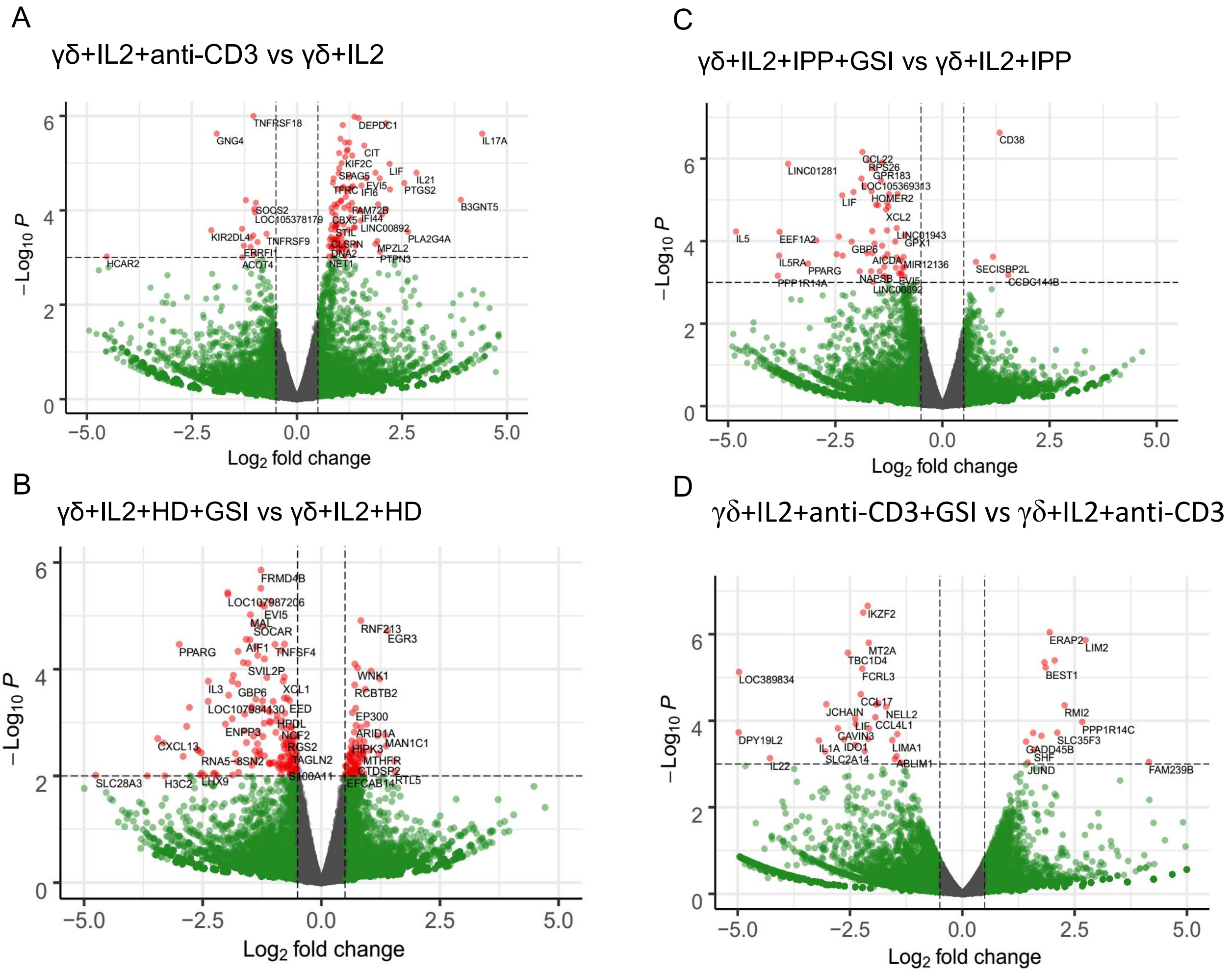

Supplementary Figure S4. Differentially expressed (DE) genes for TCR activation and Notch inhibition. A. Volcano plots showing DE upon anti-CD3 treatment of gamma-delta cells. B, C and D show DGEs upon various activation treatments coupled with GSIx-induced Notch inhibition.

Supplementary Figure 5

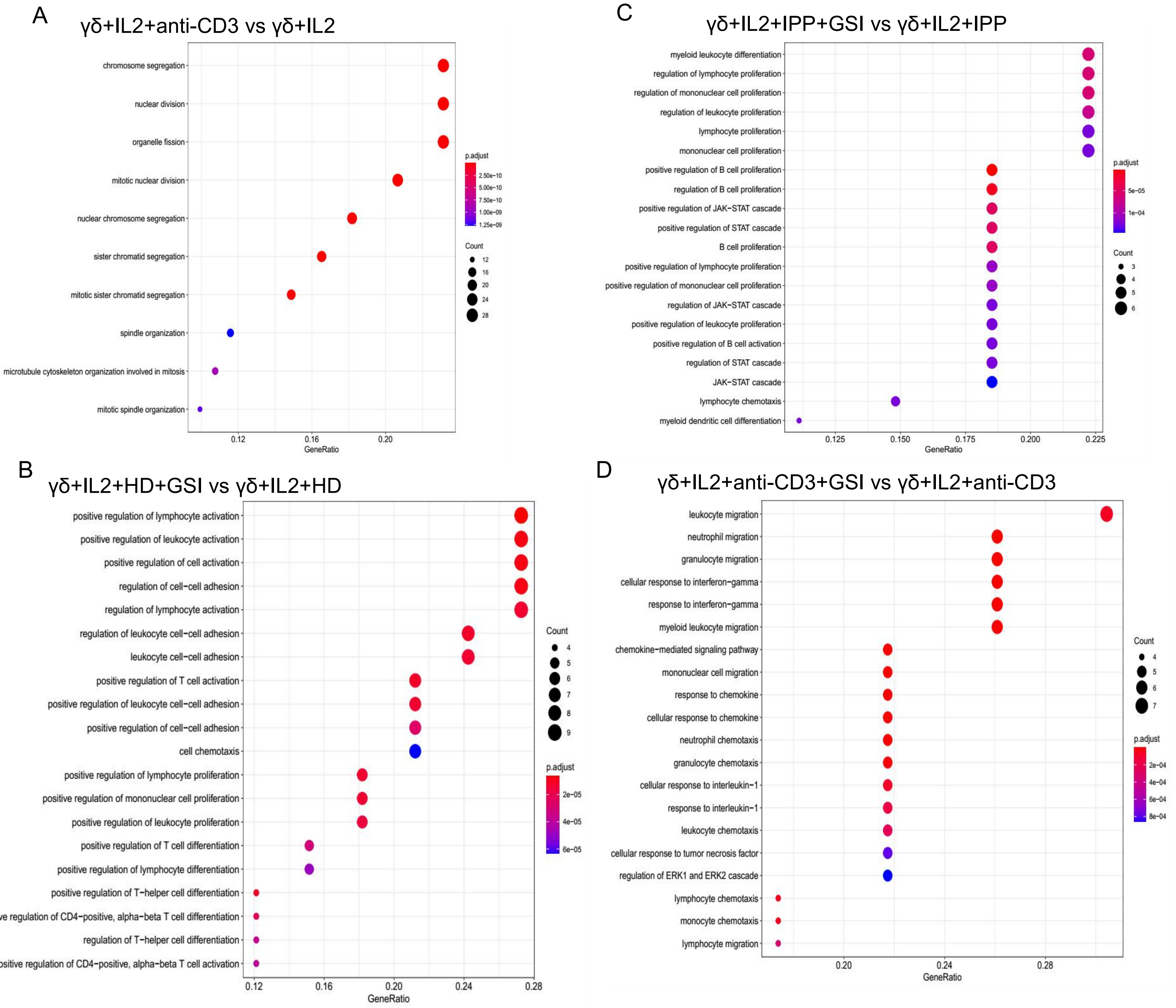

Supplementary Figure S5. Pathway enrichment upon TCR-signal activation and Notch-signal inhibition. Most significantly enriched gene ontology (GO) terms upon A. anti-CD3 activation, B. GSI treatment following HDMAPP, C. GSIx treatment along with IPP treatment and D. GSI treatment with anti-CD3 stimulation of gamma delta T cells.

Supplementary Figure 6

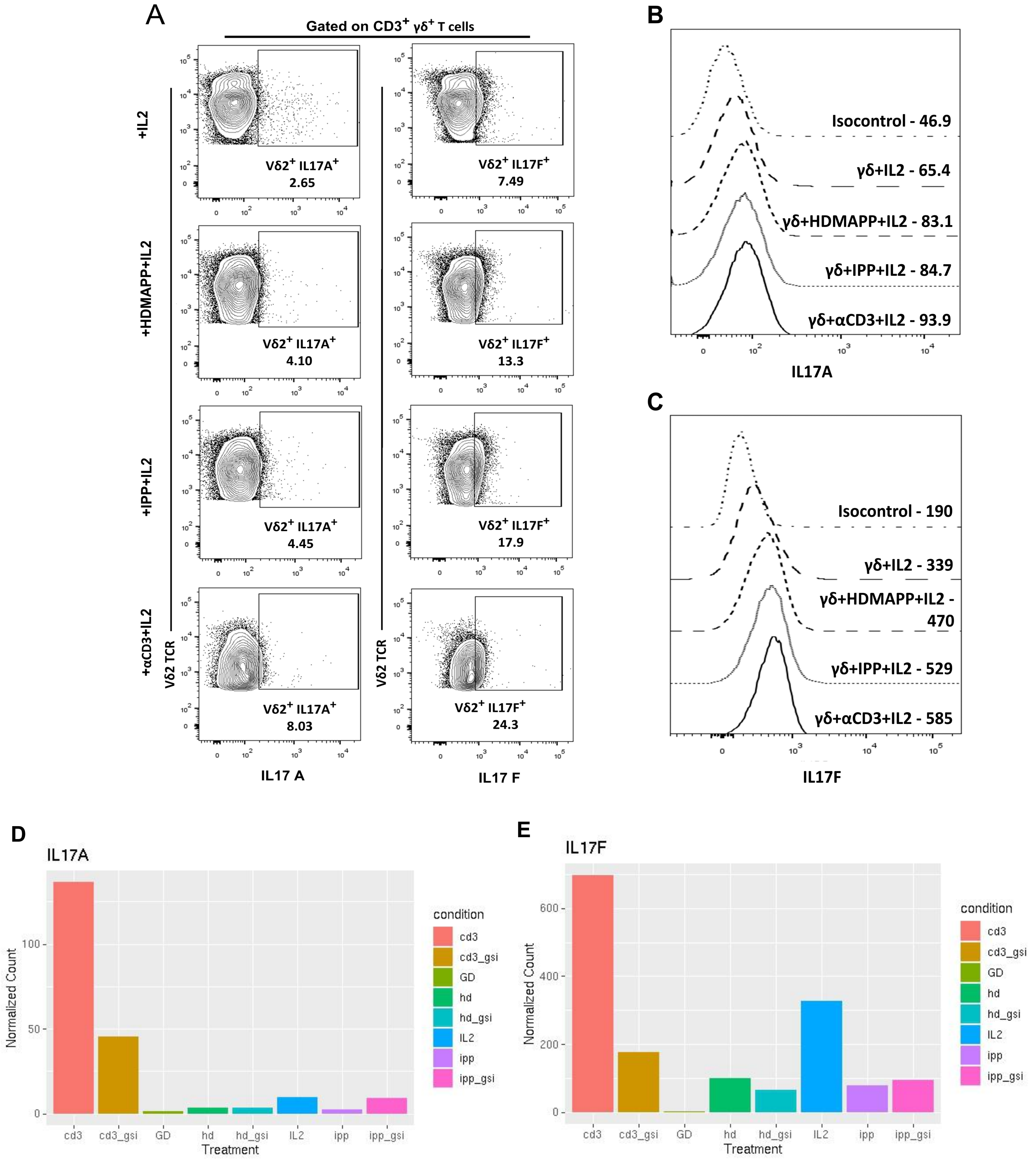

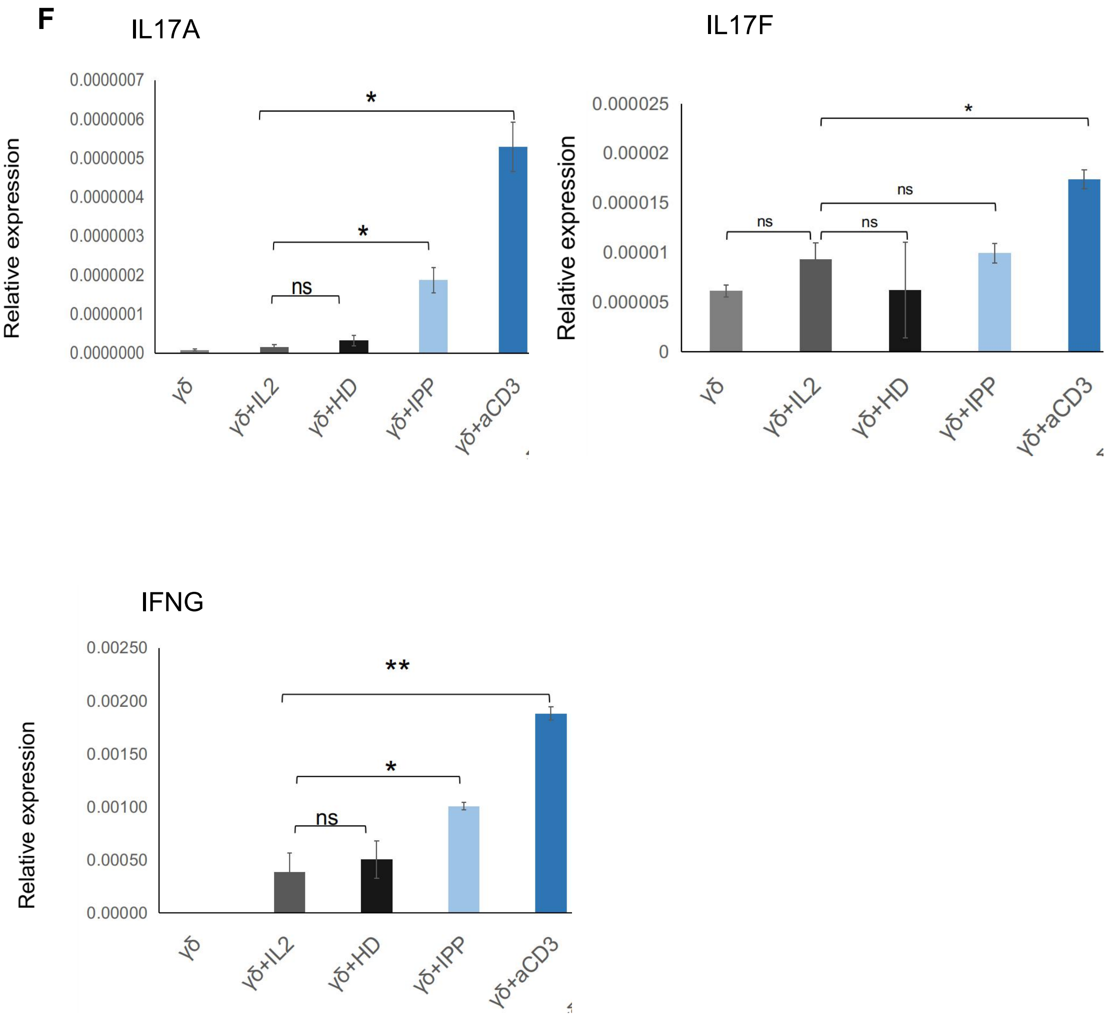

Supplementary Figure S6. Intracellular expression of IL17A and IL17F in stimulated γδ T cells. The expression of cytokines in γδ T cells isolated from peripheral blood lymphocytes of healthy individuals (n=3) and differentially stimulated were assessed by multicolor flow cytometry. A. Representative figure showing intracellular expression of cytokines, IL17A and IL17F, gated on CD3+ Vδ2-TCR+ γδ T cells in IL2, HDMAPP+IL2, IPP+IL2, or anti-CD3+IL2 stimulated γδ T cells after 72 h culture. The values represented in the figure indicate the percentage-positive population. B, C. The median fluorescence intensity (MFI) of IL17A and IL17F expression observed in differentially stimulated γδ T cells was analyzed after 72 h culture. The values in the inset represent the MFI of the respective expressions. D and E. Normalized transcript counts of IL17A and IL17F, respectively, across all activation and notch inhibited conditions compared to γδ only (GD) and IL2 treatment (n=3). F. qRT-PCR validation of IL17A, IL17F and IFNG in IL2, HDMAPP+IL2, IPP+IL2, or anti-CD3+IL2 stimulated γδ T cell . * $p<0.05$, ** $p<0.01$. Graphpad v1.2 was used to perform unpaired Students t-test.

Supplementary Figure 7

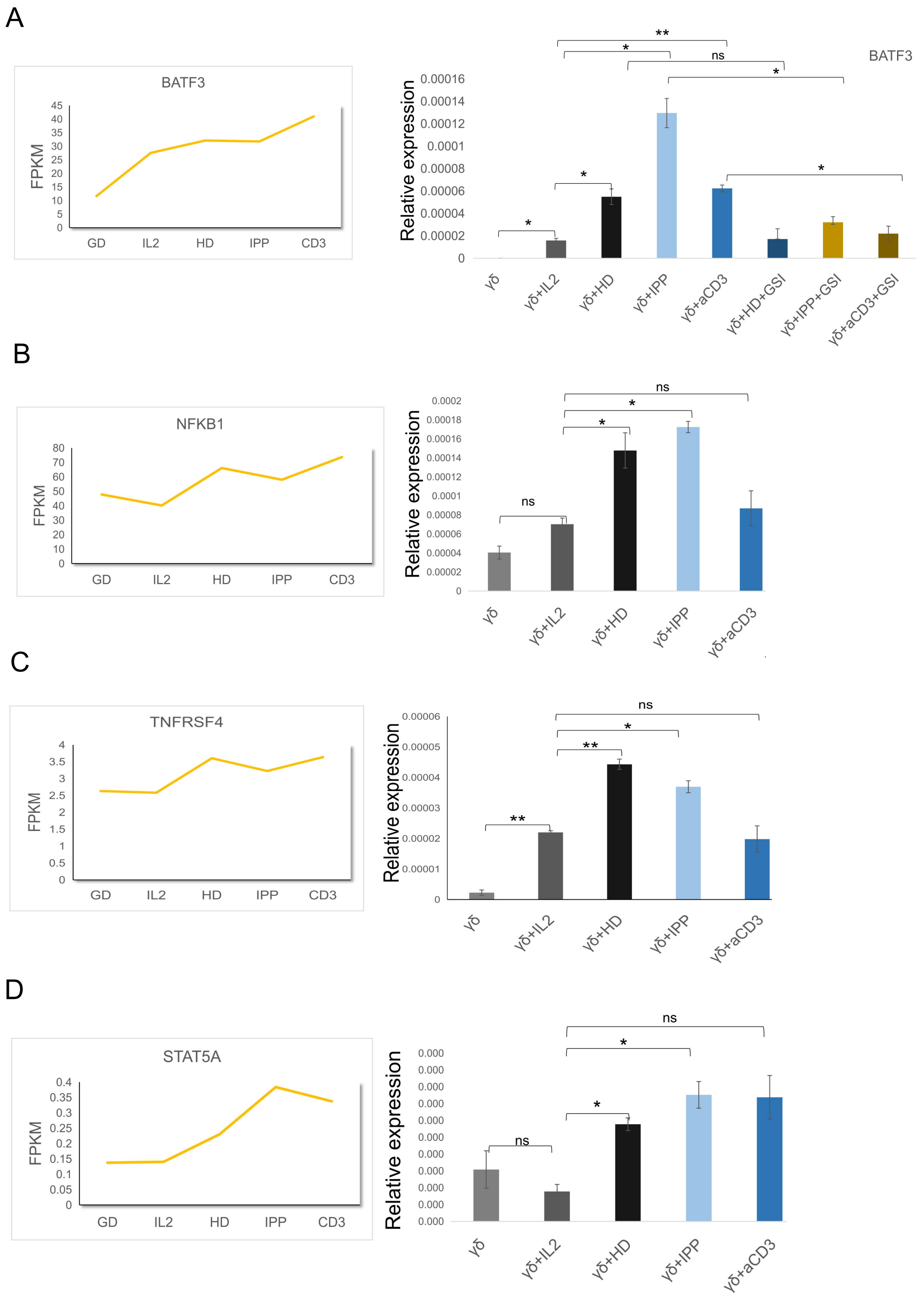

Supplementary Figure S7: Gene expression of activation induced factors: qRT-PCR validation of some of the transcription factors up-regulated upon activation via HDMAPP/IPP or anti-CD3 (n=3). A-D FPKM plots (left panel) shows the trend of differentially regulated TFs upon each activation treatment from GD only and IL2 treated GD T cells. A-D qPCR analyses (right panel) was done for the target genes, normalized to 18s rRNA transcripts in each condition. * p<0.05, ** p<0.01. Graphpad v1.2 was used to perform unpaired Students t-test.

Supplementary Figure 8

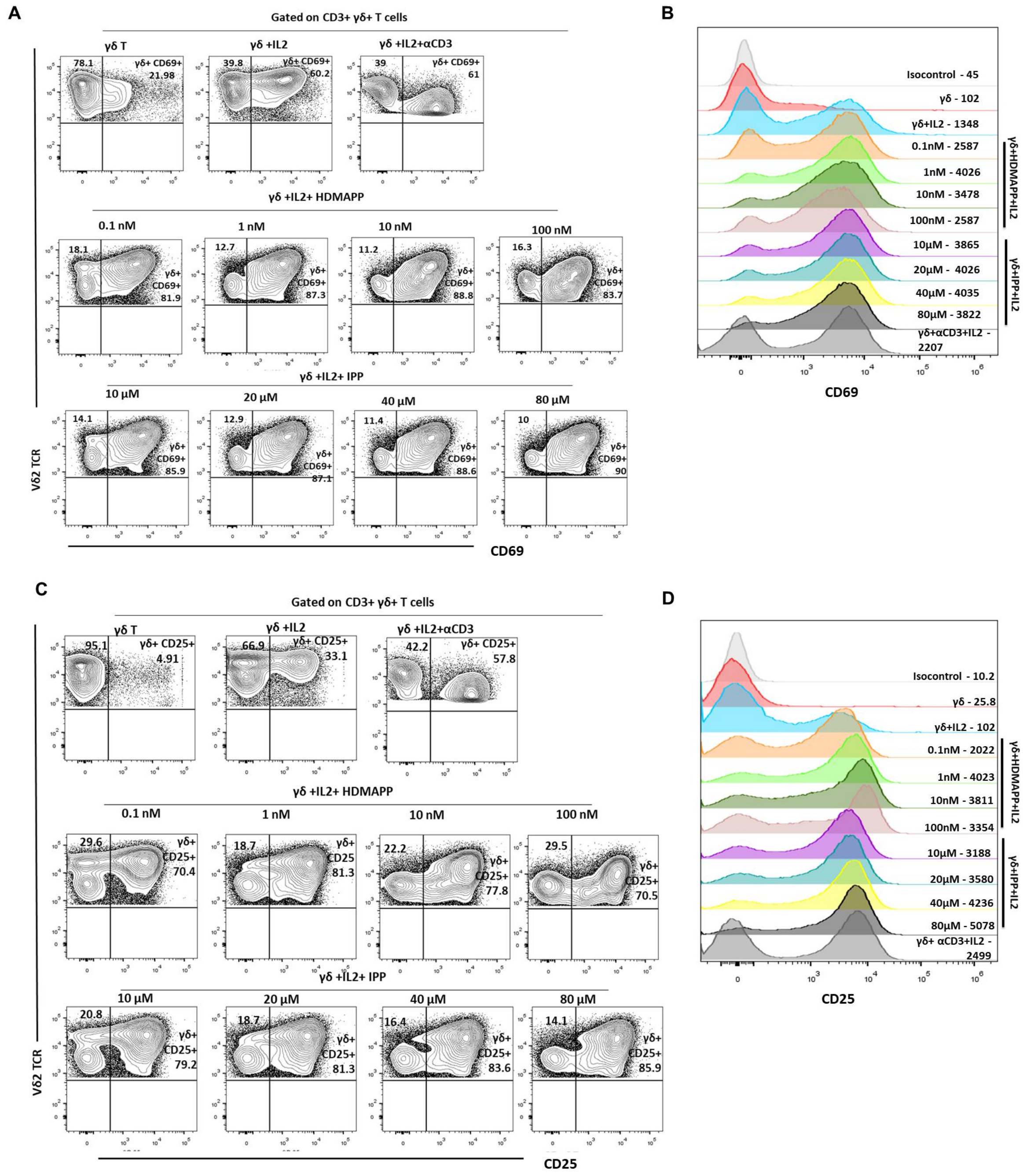

Supplementary Figure S8. Expression of activation markers CD69 and CD25 on phosphoantigen stimulated γδ T cells. γδ T cells isolated from peripheral blood of healthy individuals (n=3) were stimulated with IL2, HDMAPP+IL2 (0.1 nM-100 nM), IPP+IL2 (10 μM-80 μM), anti-CD3+IL2 (2.5 μg/mL) or kept unstimulated for 72 h. Representative figure showing the expression of CD69 (A) and CD25 (C) on γδ T cells treated with IL2, HDMAPP+IL2, IPP+IL2, anti-CD3+IL2 or kept unstimulated for 72 h. The values in the figure represent the percent positive population of $V\delta2^{+}$ $CD69^{+}$ and $V\delta2^{+}$ $CD25^{+}$, respectively. B, D. The median fluorescence intensity (MFI) of CD69 and CD25 expression on differentially stimulated γδ T cells are shown.

Supplementary Figure 9

Supplementary Figure 9

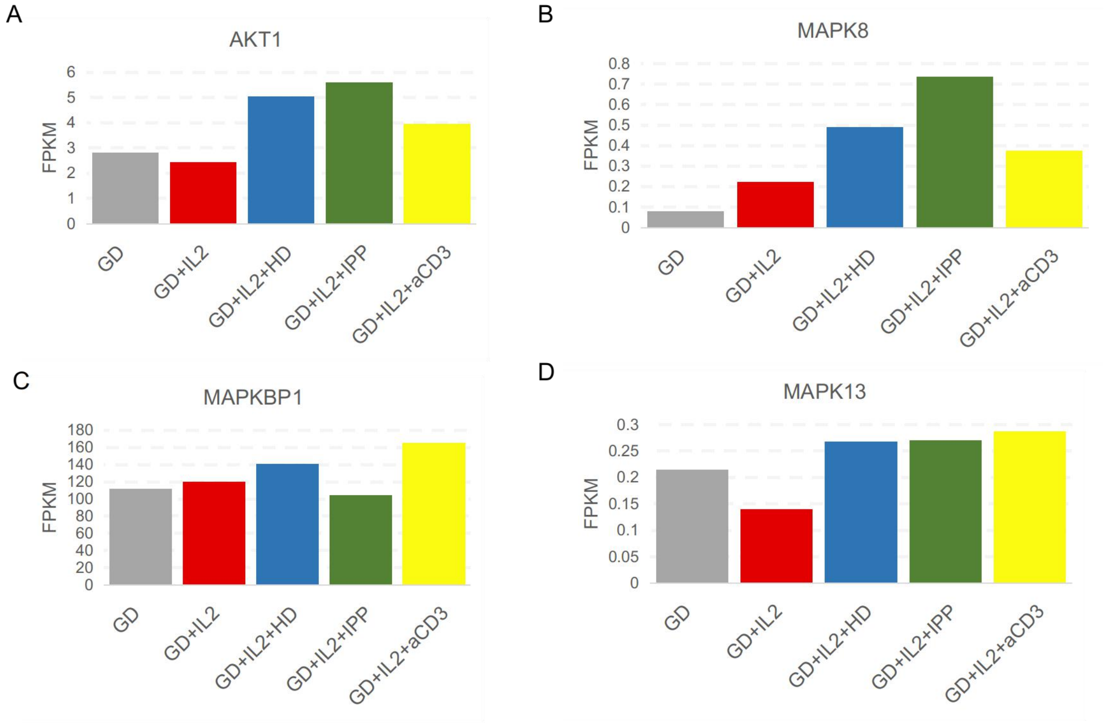

Supplementary Figure S9: A-D. PI3K/AKT and ERK/MAPK pathway key genes were plotted as FPKM values for γδ only (GD),IL2, IL2+HDMAPP, IL2+IPP and IL2+anti-CD3 samples (n=3).
